# Attention extends beyond single words in beginning readers

**DOI:** 10.3758/s13414-020-02184-y

**Published:** 2020-10-29

**Authors:** Joshua Snell, Christophe Cauchi, Jonathan Grainger, Bernard Lété

**Affiliations:** 1 Aix-Marseille Université, Marseille, France; 2 Centre National de Recherche Scientifique, Paris, France; 3 Vrije Universiteit Amsterdam, Amsterdam, the Netherlands; 4 Université Lumière de Lyon 2, Lyon, France

**Keywords:** Reading, Attention, Development

## Abstract

A common notion is that during the first stages of learning to read, attention is narrowly focused so as to encompass only a single or a few letters. In skilled adult readers, however, attention extends beyond single words. The latter is evidenced by faster recognition of words that have many letters in common with surrounding words, along with correlations between such integration effects and measures of attention. These premises suggest that the distribution of attention gradually increases as a function of reading skill, and that this progression can be mapped by measuring spatial integration effects across the course of reading development. The latter was undertaken in the present study, in which we employed the flanker paradigm combined with the lexical decision task. Children in grades 1―6 (N = 113) were shown central target words flanked by various types of orthographically related and unrelated flanking stimuli. Against expectations, significant effects of flanker relatedness on word recognition speed were found in the youngest children, and this effect was not modulated by reading age. Our results challenge the notion that attention is focused on single letters in beginning readers, and instead suggest that, from the earliest stages of reading development, orthographic processing can extend beyond single words.

## Introduction

To what extent does experience determine the amount of information readers can extract from the visual field in a single glance? By most accounts of reading development, beginning readers are thought to apply a strictly sequential processing strategy, both in terms of letter-in-word processing as well as word-in-sentence processing. The main theoretical motive here is processing capacity: the untrained system has to spend more effort recognizing letters and words, and would therefore allow for processing of limited portions of information at once (e.g., Ans, Carbonnel, & Valdois, 1998). Some empirical observations seem to attest to this conception. Beginning readers process sentences with more fixations and shorter saccades than do skilled readers (Rayner, 1986). Additionally, when allowing only five characters to be visible at once (the so-called moving window technique), this inflicts a cost of approximately 70% in skilled readers’ reading speed, while inflicting a mere 30% cost in beginning readers (Rayner 1986; see also Blythe, Liversedge, Joseph, White, & Rayner, 2009; Sperlich, Schad, & Laubrock, 2015).

Many would agree also that sequential processing of letters within words is eventually replaced by parallel letter processing (e.g., Adelman, Marquis, & Sabatos-DeVito, 2010). Indeed, research has shown that parallel letter processing even extends well beyond the fixated word in skilled adult readers, with words being recognized faster if they have many letters in common with adjacent words (Dare & Shillcock, 2013; Grainger, Mathôt, & Vitu, 2014; Inhoff, Radach, Starr, & Greenberg, 2000; Snell, Vitu, & Grainger, 2017; Snell, Bertrand, Meeter, & Grainger, 2018a; Snell, Bertrand, & Grainger, 2018b). In further revealing differences between beginning and skilled readers in this regard, Khelifi, Sparrow, and Casalis (2017) found that the recognition of foveal target words was facilitated by parafoveally presented repetition primes (relative to unrelated primes) in fifth graders and adults but not in third graders. It therefore seems reasonable to claim that readers’ processing span increases with experience.

However, more detailed accounts of how the processing span evolves during reading development cannot be constructed without resolving some theoretical ambiguities. As has been recognized by others (e.g., Kwon, Legge, & Dubbels, 2007), it is not yet clear whether the processing span is constrained by purely linguistic factors, low-level visual operations, or both. Kwon et al. (2007) provided evidence for differences in visual processing across the course of reading development. They found that the recognition of single letters in peripheral regions of vision (relative to foveal letter recognition) improved with increasing age and correlated with reading speed. On the other hand, Rayner (1986) evidenced the involvement of linguistic factors, as the processing span of fourth-grade readers, when provided with easier age-appropriate texts, approximated that of adult readers. Relatedly, employing the moving window technique, Sperlich et al. (2015) found that increasing limitations on the availability of parafoveal information hampered reading more severely in third-graders than second-graders, while such window size effects did not differ between second- and first-graders. This led the authors to conclude that the perceptual span in developing readers is mainly contingent on the automation of basic word recognition processes (e.g., orthographic processing). Indeed, some have expressed the belief that beginning and skilled readers extract equal amounts of visual information from the visual field during each fixation, but that skilled readers have more rapid access to letter and word codes (e.g., Jackson & McClelland, 1979; Neuhaus, Foorman, Francis, & Carlson, 2001). Such reasoning seems to dissociate between at least two types of processing span: one representing low-level visual processing (conceivably up to the level of feature detection) that would remain stable from the first moments of learning to read, and the other being a span of orthographic and lexical processing that would increase in size with experience.

Following a synthesis of the above, to claim that the perceptual span is smaller in beginning readers (e.g., Khelifi et al., 2017; Kwon et al., 2007) might be overly simplistic. Rather, it appears that there exist more intricate differences between beginning and skilled readers with respect to the amount of information they extract from the visual field. Visuo-spatial attention may be the common denominator here. It is already known that how attention is distributed across the visual field is strongly determined by tasks and contexts: for instance, whereas in the classical Eriksen flanker task using non-linguistic stimuli (Eriksen & Eriksen, 1974), attention was found to be biased to the left (Harms & Bundesen, 1983); in flanker tasks that employ word stimuli, attention is biased to the right (Snell & Grainger, 2018), analogous to the attentional bias observed during sentence reading (Rayner, 1998). Bearing this in mind, one way to reconcile reports that parafoveal letter detection is worse in beginning readers (Khelifi et al., 2017; Kwon et al., 2007) with reports that the perceptual span of beginning readers can under the right circumstances match that of skilled readers (Rayner, 1986), is to hypothesize that the artificial nature of the tasks employed by Kwon et al. and Khelifi et al. (respectively trigram identification and sequential presentation of words at different locations) affected the attentional distribution in beginning readers differently to that of skilled readers. When engaged in “real” reading, however, the attentional distribution may be quite similar between beginning and skilled readers, with linguistic difficulty determining not so much the size of the processing span but rather the depth of processing (features, letters, or whole words) across the span.

### The present study

Taken together, it is clear that the field has not yet converged towards a concrete account of the processing span during reading development. Aiming to provide a stride in the right direction, here we report an investigation into the distribution of visuo-spatial attention in young readers. The central objective of this study was to provide a direct test of the common notion that attention is narrowly distributed in beginning readers and widens with experience.

We built on recent lines of research in which we established a fairly simple way to track the distribution of attention. Snell, Mathôt, Mirault, and Grainger (2018c) found that aforementioned orthographic spatial integration effects, whereby words are recognized faster when surrounded by the same words (e.g., *rock rock rock*) compared to different words (*step rock step*) (Dare & Shillcock, 2013; Snell et al., 2017, 2018a, 2018b), correlate with the distribution of covert (i.e., without looking) visual attention. They made use of the principle that pupillary light responses (whereby the pupils dilate or constrict as the result of observing dark or bright things, respectively) are triggered not just by the things we look at directly, but also by the things we attend to covertly (for a review, see Mathôt & van der Stigchel, 2015). Implementing this in a flanker paradigm wherein participants made lexical decisions about central target words surrounded by words either on the left and right or above and below the target, Snell et al. (2018c) found that pupil size was contingent with the brightness of flanking words left and right of the target, but not flanking words above and below the target. Perfectly in line with this asymmetry, recognition speed was influenced by the orthographic relatedness of flanking words left and right of the target, but not flanking words above and below the target; this suggests that attention is indeed allocated to the stimuli involved in orthographic integration effects (see also Snell, Meade, Meeter, Holcomb, & Grainger, 2019, for electro-encephalographic evidence against pre-attentive accounts of orthographic integration effects).

Due to its methodological simplicity, the flanker paradigm provides the perfect means to track the attentional distribution in beginning and skilled readers: specifically, attention can be claimed to extend beyond single words in those readers who show an influence of the orthographic relatedness of flankers on target recognition speed.

Naturally, one drawback of the flanker paradigm is that it is unlike natural (sentence) reading. Should we find here, for instance, that portions of attention are allocated to the parafovea, it remains to be seen whether beginning readers exhibit similar patterns when reading sentences. Interestingly, however, studies with adult readers have thus far shown that attention, as observed through orthographic integration effects, operates quite similarly between these two settings (Dare & Shillcock, 2013; Snell et al., 2017; Snell & Grainger, 2018). For instance, the rightward attentional bias inherent to sentence reading also occurs in the flanker paradigm, reflected by a stronger impact from rightward word flankers (Snell & Grainger, 2018). Such findings lead to the belief that a flanker paradigm with words does to a certain degree engage participants in “real” reading – at least with respect to the attentional distribution (see Snell & Grainger, 2019a, for a more detailed outline of this rationale).

One must nonetheless take note of some methodological challenges integral to the employment of this paradigm with young populations. In previous implementations of this paradigm, brief (150-ms) stimulus durations were key to being able to argue for parallel letter and word processing. Given that this is barely enough time to recognize single words (with estimations of average word recognition speed being in the 150- to 250-ms range; e.g., Rayner, 1998), the flankers must be processed *during* rather than *after* target processing if they are to have any influence on target recognition. However, considering that word recognition is much slower in beginning readers (e.g., Rayner, 1986, 1998), 150 ms is not sufficient for this population to perform the lexical decision task above chance level. Indeed, during pilot testing, we found that age influenced the minimal duration needed to make lexical decisions with a reliability above 60%. Specifically, our least proficient participants (N = 44, sampled from grades 1 and 2) required a stimulus duration of 300 ms, while 250 ms sufficed for children of intermediate proficiency (N = 69, sampled from grades 3, 4, 5, and 6) (see also Seabra, Dias, Mecca, & Macedo, 2017). This difference may be regarded as a potential confound when interpreting interactions between flanker relatedness effects and reading age. Therefore, in addition to testing for modulatory effects of reading age in the entire sample, we also carried out the same analyses on the subset of participants for whom we used a consistent 250-ms presentation time.

In addition to the various flanker conditions outlined below, we also included a no-flanker condition that allowed us to investigate the impact of the presence of surrounding stimuli per se. The mere establishment of flanker relatedness effects would have allowed us to claim that attention is widely distributed in our population; but we would not have been able to determine whether that wide attentional distribution is the default state or rather the result of surrounding stimuli inevitably drawing processing resources away from the fixated word. If we were to find that effects of flanker presence are modulated by reading age, this would allow us to formulate an account of the relationship between reading experience and attentional control.

## Methods

### Participants

One hundred and thirteen children from a public elementary school (grades 1―5; N = 99) and from a public secondary school (grade 6; N = 14) in Lyon, France, were tested at the end of their school year in June. These children were included on the basis of having a reading proficiency matching that of their grade (pre-tested with the Alouette reading test; Cavalli et al., 2018; Lefavrais, 1967), and on the basis of having an accuracy score >60% in the main experiment. All participants were native French speakers with normal or corrected-to-normal vision. Informed consent was provided by the participant’s caregivers prior to experimentation. Ethical approval for this study was granted by the *Comité de Protection des Personnes SUD-EST IV* (No. 17/051) in Lyon.

### Stimulus selection and materials

One hundred and twenty word pairs were selected from the Manulex lexical database (Lété, Sprenger-Charolles, & Colé, 2004). All words were four letters long, were classified as Grade 1 in the Manulex corpus (meaning that these words are regularly encountered by beginning readers), and contained no diacritics. The mean frequency of these words was 2.55 log parts per million (ppm) (range 0.78—3.94 log ppm). The word set included verbs, adjectives, participles, pronouns, adverbs, and auxiliaries. None of the words in each pair were orthographically or semantically related, and these pairs of words were used to generate the target words and the unrelated flanker words. A set of 120 pseudoword pairs were generated with Wuggy (Keuleers & Brysbaert, 2010). These were used to induce the lexical decision task, and were thus not included in our analyses.

### Design and statistical power estimation

We employed six conditions that can be subdivided into two independent manipulations of flanker relatedness (meaning the study did not have a fully crossed design). The first three conditions were adapted from the study of Grainger et al. (2014), who used bigram flankers in a repetition condition (e.g., *ro rock ck*), a condition that switched bigram flankers (*ck rock ro*) and an unrelated bigram flanker condition (*st rock ep*). The remaining three conditions were adapted from the study of Snell and Grainger (2018), which used whole-word flankers: a repetition condition (*rock rock rock*), an unrelated condition (*step rock step*), and a no-flanker condition. An overview of all experimental conditions is provided in Table 1. We verified that none of the targets formed a new word when combined with one of its bigram flankers (which could otherwise have biased lexical decisions).

**Table 1:** Stimuli across experimental conditions. Although these examples are in English, we used French words (and also note that pseudoword stimuli were used as filler trials)

	Word	Pseudoword
Repetition bigrams	ro rock ck	zo zock ck
Switched bigrams	ck rock ro	ck zock zo
Unrelated bigrams	st rock ep	st zock el
Repetition words	rock rock rock	zock zock zock
Unrelated words	step rock step	stel zock stel
No flankers	rock	zock

With a Latin-square design we ensured that all items (i.e., both words and pseudowords) were shown in all conditions, but only once per participant. The total of 240 trials was presented in randomized order.

The total of 2,260 measurements per experimental condition (pseudoword trials excluded) meets the recommendation of Brysbaert and Stevens (2018) for having abundant statistical power (their recommendation being 1,600 measurements per condition). We additionally estimated statistical power ad hoc based on data from the study of Snell and Grainger (2018). In our previous study, in which we also employed repetition flankers and unrelated flankers, we observed a mean 36-ms difference in response time based on 2,000 measurements per condition, with a Cohen’s *d* = 0.30. Statistical power was estimated using simulations with the *simR* package (Green & MacLeod, 2016) in the R computing environment. When drawing 200 random samples, and using the linear mixed model structure reported in Snell and Grainger (2018), a significant effect was returned 96.38% of the time; hence an estimated power of 0.96. Given that we performed a higher number of measurements per condition in the present study, we expect to have had abundant statistical power. All data are available at https://osf.io/9jkt6/.

### Apparatus and software

The stimuli and experimental design were implemented with OpenSesame (Mathôt, Schreii, & Theeuwes, 2012). Stimuli were presented on an HP ProBook 640 G2 monitor calibrated in 18-in. (1,366 × 768 px, 80 Hz). Participants were seated at a 60-cm distance from the display. Stimuli were displayed in lower case, Courier New font, black color on light grey background. Each character space subtended 0.35° of visual angle.

### Procedure

**Fig. 1:**
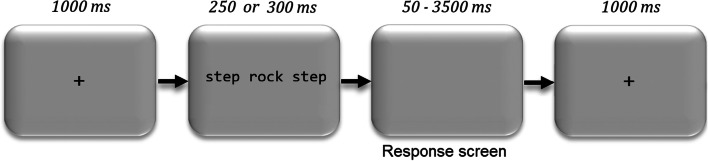
Procedure used in the Experiment. Targets and flankers were presented for a brief duration of either 250 or 300 ms, depending on the participant’s reading age. The size of stimuli relative to that of the display is exaggerated in this example

The trial procedure is shown in Fig. 1. Each trial started with a 1,000-ms centralized fixation cross. The target and flanking stimuli were then presented for a duration depending on the participant’s grade (300 ms for grades 1 and 2; 250 ms for grades 3 and beyond). Participants had a maximum of 3,500 ms to make their lexical decision with a right- (“*m*”) or left-handed (“*q*”) button press (AZERTY keyboard layout) for respectively word and pseudoword targets. The response was followed by a 1,000-ms empty blank screen preparing the next trial. The trials were subdivided into ten blocks of 24 trials. To avoid fatigue, a break was offered in between blocks. The total duration of the task was about 20 min.

## Results

As noted under *Participants*, our 113 participants all had an accuracy score > 60%. For the analysis of response times (RTs), we excluded incorrectly answered trials (21.40% of trials) and trials with a response time (RT) beyond 2,500 ms (1.45% of the correctly answered trials). For the analysis of errors, the latter criterion led to the exclusion of 4.39% of trials.

Although pseudoword trials were merely employed as filler trials to induce the task (and are therefore not included in our analyses of interest), we did assess task performance in pseudoword trials to verify that children performed the lexical discrimination task above chance. Error rates (ERs) for pseudoword trials were analogous to those observed in word trials across all levels of proficiency (overall, ER_word_ = 0.21, ER_pseudo_ = 0.26).^1^

Data were analyzed using linear mixed-effect models (LMMs) with flanker condition and reading age as independent variables, and items and participants as crossed random effects. We used models with the maximal random structure that successfully converged. For the analyses of RTs, this was a model that included by-item and by-participant random slopes for the condition factor, alongside random intercepts. The analysis of errors was performed with a model that only included by-item and by-participant random intercepts. We report *b*-values, standard errors (SEs), and *t*-values (RTs) or *z-*values (errors), with *t*- and *z*-values beyond |1.96| deemed significant.

Below, results are reported for two sets of conditions. The first section presents results for the bigram flanker conditions. The second section presents results for the whole-word and no-flanker conditions. As noted in the *Introduction*, direct tests of an interaction between flanker relatedness and reading age were performed both on the entire sample, as well as on the 69 most proficient children (sampled from grade 3 and beyond) for whom we used a consistent 250-ms presentation duration. Prior to analyzing RTs, we applied a log-transformation to bring the data in line with the normality assumption.

### Bigram flankers

**Fig. 2:**
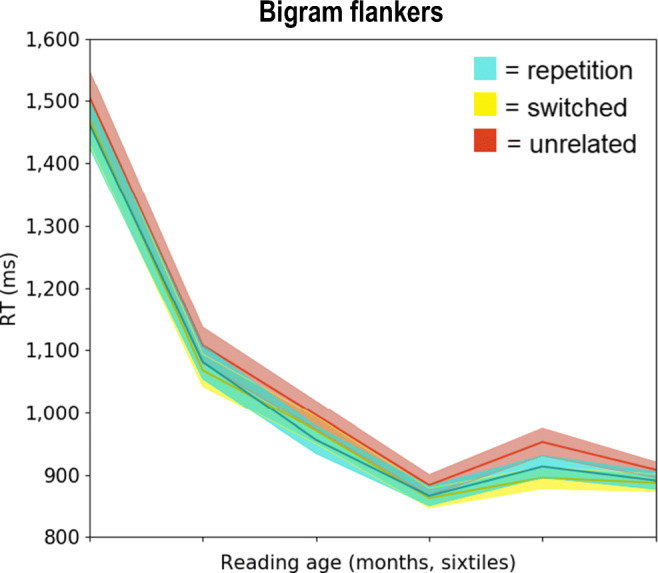
Average response times (RTs) for the bigram flanker conditions as modulated by reading age. Data were divided into six quantiles, sorted by reading age (least to most proficient). Shaded areas around the curves depict standard errors. Note that RTs in the repetition (blue) and switched (yellow) conditions largely overlap

**Fig. 3:**
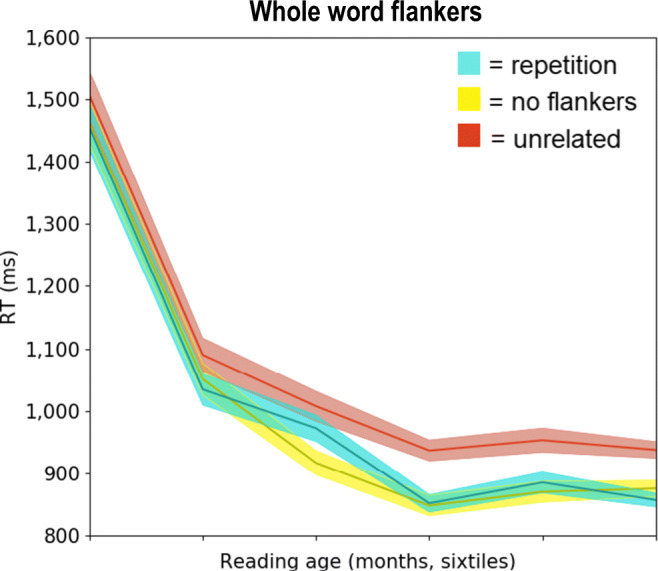
Average response times (RTs) for the whole-word and no-flanker conditions as modulated by reading age. Data were divided into six quantiles, sorted by reading age (least to most proficient). Shaded areas around the curves depict standard errors

Figure 2 shows raw RTs for the bigram flanker conditions across the proficiency range. Note that although Figs. 2 and 3 show raw RTs for illustrational purposes, statistical analyses were performed on log-transformed RTs.

We observed a main effect of Reading Age, with better performance of more proficient participants (RTs: *b* = -0.006, SE = 0.001, *t* = -5.65; errors: *b* = -0.02, SE = 0.004, *z* = -6.22). Replicating findings from previous research, the flankers also had a significant effect on performance, with repetition flankers yielding faster responses than unrelated flankers (*b* = -0.04, SE = 0.001, *t* = -4.29) and fewer errors (*b* = -0.44, SE = 0.08, *z* = -5.30). Like the intact-order flankers, the switched flanker condition yielded faster responses than the unrelated flanker condition (*b* = -0.04, SE = 0.01, *t* = -4.56) and fewer errors (*b* = -0.17, SE = 0.08, *z* = -2.09). No difference in RT was observed between the intact and switched flanker conditions (*b* = 0.01, SE = 0.01, *t* = 0.86), although fewer errors were made in the presence of intact flankers compared to switched flankers (*b* = -0.27, SE = 0.08, *z* = -3.24).

Of crucial importance to the central question of this paper – whether attention extends beyond single words in beginning readers – is the fact that effects of Flanker Relatedness were also present in the youngest, least proficient children. Isolating the 20 least proficient participants and contrasting repetition versus unrelated flankers, we observed a marginally significant effect in RTs (*b* = 0.05, SE = 0.03, *t* = 1.95) and a significant effect in errors (*b* = 0.55, SE = 0.17, *z* = 3.25).

Reading age was not found to modulate the flanker effect, both when analyzing the entire sample (RTs, *b* = 3*10^-4^, SE = 4*10^-4^, *t* = 0.62; in errors, *b* = 0.006, SE = 0.004, *z* = 1.37) and when analyzing the subset of children for whom we used consistent 250-ms stimulus durations (N = 69; RTs, *b* = 3*10^-4^, SE = 6*10^-4^, *t* = 0.50; errors, *b* = 0.006, SE = 0.007, *z* = 0.94).

### Whole-word flankers

Figure 3 shows raw RTs for the two whole-word flanker conditions (repetition vs. unrelated) and the no-flanker condition. The main effect of reading age was again expressed both in log-transformed RT scores (*b* = -0.006, SE = 0.001, *t* = -5.65) and in errors (*b* = -0.02, SE = 0.003, *z* = -6.09). Compared to repetition flankers, responses were again slowed by unrelated flankers, *b* = 0.07, SE = 0.008, *t* = 8.62. This difference was also expressed in the error rate, *b* = 0.37, SE = 0.08, *z* = 4.41. The no-flanker condition yielded better performance than repetition flankers (RTs: *b* = -0.12, SE = 0.05, *t* = -2.55; errors: *b* = 0.07, SE = 0.09, *z* = 0.75) and than unrelated flankers (RTs: *b* = -0.18, SE = 0.05, *t* = -3.61; errors: *b* = -0.31, SE = 0.08, *z* = -3.65).

Isolating the 20 least proficient readers, we again observed effects of flanker relatedness in the two whole-word flanker conditions (repetition vs. unrelated): RTs: *b* = 0.07, SE = 0.03, *t* = 2.81; errors: *b* = 0.33, SE = 0.17, *z* = 1.89. Similar to our observations for bigram flankers, reading age was not found to modulate whole-word flanker effects, both when analyzing the entire sample (RTs: *b* = 2*10^-4^, SE = 4*10^-4^, *t* = 0.37; errors: *b* = 0.004, SE = 0.004, *z* = 0.85), and when isolating the 69 readers from grade 3 and beyond (RTs: *b* = 4*10^-4^, SE = 6*10^-4^, *t* = 0.72; errors: *b* = 0.004, SE = 0.007, *z* = -0.49).

As noted in the *Introduction*, the no-flanker condition allowed us to investigate whether the impact of the presence of surrounding stimuli per se is modulated by reading experience. With the no-flanker condition as reference, a contrast against the repetition flanker condition revealed a significant interaction between Flanker Presence and Reading Age, whereby the adverse effect of the presence of flankers, as reflected in RTs, diminished with increasing reading experience, *b* = -0.001, SE = 4*10^-4^, *t* = -2.26. This effect was not reflected in the error rate, however (*b* = 0.006, SE = 0.005, *z* = 1.36). A marginally significant interaction between Flanker Presence and Reading Age was observed in RTs when isolating the no-flanker and unrelated flanker conditions (RTs: *b* = 0.001, SE = 4*10^-4^, *t* = -1.85; errors: *b* = 0.002, SE = 0.004, *z* = 0.58).

Lastly, we investigated whether differences in word familiarity across our population sample (older children being more familiar with certain stimuli than younger children) may have contributed to our effects. If this were indeed possible, then our key effect of orthographic overlap should similarly be modulated by word frequency in all participants. Re-running our model with log-frequency added as a variable alongside our condition factor, we established a main effect of frequency with better overall performance for highly frequent words (RTs: *b* = -4.87*10^-5^, SE = 1.73*10^-5^, *t* = -2.82, errors: -2.28*10^-4^, SE = 9.27*10^-5^, *z* = -2.46), but, importantly, no interaction between frequency and flanker relatedness (RTs: *b* = 5.31*10^-6^, SE = 1.75*10^-5^, *t* = 0.30; errors: *b* = 1.36*10^-4^, SE = 1.07*10^-4^, *z* = 1.27). We therefore deduce that differences in word familiarity across participants need not be taken into account in the interpretation of (the absence of) interactions between flanker relatedness and reading age.

## Discussion

While it has been generally assumed that the processing span is smaller in beginning readers (Ans et al., 1998; Khelifi et al., 2017; Rayner, 1998), an aggregate of various lines of research suggests that this assumption is subject to some theoretical ambiguity. Specifically, whereas some studies point to a reduced visual processing span in beginning readers (e.g., Khelifi et al., 2017; Kwon et al., 2007), other studies suggest that the span of visual processing is more or less stable across the course of reading development, and that it is instead linguistic factors that determine how much information the beginning reader can process within a given interval (e.g., Blythe et al., 2009; Rayner, 1986). Aiming to clarify matters, in the present study we used a flanker paradigm combined with the lexical decision task in order to determine the attentional distribution in beginning readers.

Previous research has shown that orthographic spatial integration effects, whereby words are recognized faster when surrounded by related than by unrelated letters (Dare & Shillcock, 2013; Inhoff et al., 2000; Snell et al., 2017, 2018a, 2018b), are driven by attention (Snell et al., 2018c, 2019; Snell & Grainger, 2018). The fact that such effects are robustly observable in adult readers suggests that attention extends beyond single words in this population. We invoked the same logic in the present study: if the attentional distribution is confined to single letters (or at least single words) at the start of reading development, and widens as a function of reading experience, then this should be reflected in an onset of flanker relatedness effects sometime along the course of reading development – here examined in readers from grades 1–6.

The results of this study are quite unlike our prior expectations: effects of flanker relatedness were observed in the youngest, least proficient readers, and did not change through increased reading proficiency. Crucially, the stimulus durations used in each respective grade were barely sufficient to recognize single words (e.g., Seabra et al., 2017), which indicates that the flanking stimuli were processed in parallel with the target.^2^ These findings compel us to claim that attention extends beyond single words in the youngest readers.

We also established interaction effects between reading experience and flanker presence, with the adverse effect of the presence of surrounding stimuli decreasing as experience increases. This pattern is best understood in light of the recent study of Snell and Grainger (2018), who found that surrounding stimuli affect word processing in at least two ways: firstly, through the spatial integration of orthographic information, and secondly, through bottom-up capture of covert attention (hence drawing some resources away from target word processing). The core principle is that bottom-up attentional shifts are caused by onsets at relatively limitedly attended locations. For instance, when the attentional gradient is skewed to the right, a visual onset in the left hemifield would cause an attentional shift, whereas a visual onset in the right hemifield would leave the attentional distribution relatively unaltered (for the location was already abundantly attended). Attentional capture, in this sense, is not characterized by the migration of all attentional resources to a single location, but rather by a shift in the skew of the attentional distribution. Hence, even if a leftward flanker captures attention, the rightward flanker and target may continue to be processed (e.g., Snell & Grainger, 2018). In the currently tested population, the younger readers may have allocated fewer processing resources to the parafovea (though enough resources were allocated there to process the flankers orthographically), resulting in stronger capture effects.

Our key finding, that beginning readers allocate attention to surrounding words, is at apparent odds with previous claims that attention is more narrowly distributed in less proficient readers (e.g., Bosse, Tainturier, & Valdois, 2007; Bosse & Valdois, 2009). In these studies, the attentional distribution was gauged by means of briefly presenting random five-letter strings and asking participants to recall, after stimulus offset, either the whole string or single letters at post-cued locations. However, as has previously been argued by Goswami (2015), it is not clear whether worse performance in such a task can be taken as evidence for an atypical attentional distribution. In the case of recalling entire strings, for instance, task performance is equally likely to depend on short-term and/or working memory (see also, e.g., Hachmann, Bogaerts, Szmalec, Woumans, Duyck, & Job, 2014). Additionally, less proficient readers may have atypical perceptual load thresholds for reasons other than attention (see White, Boynton, & Yeatman, 2019a, for a thorough attempt to dissociate between attentional and non-attentional factors).

With respect to the recall of single letters, it is important to note that the field has produced mixed evidence. Notably, Geiger et al. (2008) observed better recognition of single letters in peripheral vision in dyslexic readers compared to non-dyslexic readers – a finding that would, if anything, be indicative of a widened attentional distribution more so than a narrowed distribution. Along the same lines, Banfi, Kemény, Gangl, Shulte-Körne, Moll, and Landerl (2018) did not find differences in the visual attention span between dyslexic and non-dyslexic readers when controlling for phonological short-term memory. Generally, then, one may be compelled to conclude that reduced reading proficiency need not be contingent on a narrow processing span; and the present results attest to this conception.

On a methodological note, a shortcoming of the present study is that the presentation durations were crudely chosen based on grade (specifically, 300 ms for readers in grades 1 and 2 (N = 44), and 250 ms for readers in grades 3, 4, 5, and 6 (N = 69)) rather than on the individual participant’s reading proficiency. It is therefore possible that task difficulty varied across participants within grades. Although this does not undermine the main finding of the present study – orthographic spatial integration effects in early developing readers – we nonetheless want to make note of more elegant ways to avoid cross-subject variance in task difficulty – for instance, White et al. (2019a) recently implemented a staircase paradigm that adapted task difficulty to individual readers, thereby allowing them to dissociate between visual processing factors and attentional factors. Nevertheless, in our study reading age was consistently not found to modulate the effect of flanker relatedness, both when analyzing the entire sample and when analyzing the participants from grade 3 and beyond (hence avoiding the potential confound caused by varying stimulus durations). This suggests that the attentional distribution does not change along the course of reading development.

It may also be argued that the use of post-masks (e.g., presenting hashmarks at the locations of stimuli after stimulus offset) would provide stronger evidence for parallel processing of words (see, e.g., discussion in Snell & Grainger, 2019b; White, Boynton, & Yeatman, 2019b). Without masking, our participants might in theory have appealed to sensory and/or short-term memory buffers to process the flankers after stimulus offset. We are nevertheless of the opinion that our flanker effects – whether reliant on direct visual processes or sensory memory – provide evidence that flankers were processed while target word recognition was still ongoing. We therefore maintain that attention must be widely distributed during reading.

Certainly the present findings do not cast doubt over the existence of a processing bottleneck in developing readers, duly evidenced by shorter saccades and longer word-viewing times in this population compared to skilled readers (Rayner, 1986). Our findings do suggest, however, that the locus of this bottleneck is at a late cognitive stage, i.e., a stage beyond the level of sub-lexical orthographic processing. Thus, if there is truth to the claim that young readers have slower access to letter and word codes (e.g., Jackson & McClelland, 1979; Neuhaus et al., 2001), we should add that slow letter processing does not prompt sequential letter processing.

In a similar vein one may ask whether slow lexical access would necessitate sequential lexical access. Parallel word processing has long been considered controversial even for skilled adult readers (e.g., Reichle, Liversedge, Pollatsek, & Rayner, 2009), although recent lines of research do provide a growing body of evidence against serial word processing (see Snell, van Leipsig, Grainger, & Meeter, 2018d, for a review). One finding of particular relevance for the present work is that target word processing in the flanker paradigm is facilitated by semantically related flankers (compared to semantically unrelated flankers) in skilled adult readers (Snell, Declerck, & Grainger, 2018e). The fact that these effects occur in the absence of any orthographic similarity across targets and flankers, and while employing brief stimulus durations, suggests that lexico-semantic processing can in principle occur for multiple words in parallel in skilled readers. A future line of research that will bear much prominence, therefore, consists of testing beginning readers in a flanker paradigm that manipulates the semantic relatedness of flankers. If there indeed exists a processing bottleneck that allows for only one word to be activated at once in beginning readers, then we should observe no effects of semantic flanker relatedness in this population. We take note here of closely related work by Veldre and Andrews (2015), who found that, in sentence reading, the depth of parafoveal processing (sub-lexical vs. lexical), was modulated by reading proficiency in an adult population sample.

Lastly, although previous research has thus far shown that orthographic parafoveal-on-foveal effects in the flanker paradigm are analogous to those observed in natural sentence reading (Dare & Shillcock, 2013; Snell & Grainger, 2018; Snell et al., 2017), and that inferences about visuo-spatial attention as established in one paradigm may therefore apply to the other, it would be worthwhile to test the present population in a sentence-reading paradigm as well. Current observations lead us to predict that the speed of recognizing a word during sentence reading will be impacted by its orthographic relatedness to the upcoming word, even in the youngest readers. Note that, in this regard, relevant studies have already been conducted by Tiffin-Richards and Schroeder (2015b) and Pagán, Blythe, and Liversedge (2016). In samples of 8- to 9-year-old readers, Pagán et al. (2016) and Tiffin-Richards and Schroeder (2015b) observed that reading was disrupted if an upcoming (parafoveal) word contained a pair of transposed letters (e.g., *tsep*, which would be replaced by *step* once the eyes moved to the word). This suggests, in line with our own results, that orthographic processing extends beyond single words in beginning readers.

In conclusion, our results provide evidence that the span of orthographic processing, driven by attention, is not contingent on reading experience. Instead, attention extends beyond single words even in the earliest stages of reading development.

## Footnotes

1 Note that no effects of flanker relatedness were found in the pseudoword trials: contrasting repetition versus unrelated bigram flankers, *b* = 0.004, SE = 0.01, *t* = 1.20; contrasting repetition versus unrelated whole-word flankers, *b* = 0.01, SE = 0.01, *t* = 1.39.

2 Given the absence of eye-position measurements in the present study, one may be compelled to contend that children could have fixated the flankers as well as the target, and that therefore these effects weren’t driven by widespread attention but rather by very rapid serial processing. However, this is very unlikely: our prior research has shown that readers do not move their eyes to the flankers when the presentation duration is shorter than the average fixation duration (Snell et al., 2018c). Rayner (1986) established that fixation durations in beginning readers are well over 300 ms, and a later study of Tiffin-Richards and Schroeder (2015a) measured an average single fixation duration of 348 ms for short, high-frequency words in a large sample of second-grade children. Children thus had insufficient time to execute multiple fixations in the present study. Alternatively, one may argue that effects were caused by children fixating flankers *instead of* – rather than in addition to – the target. But this does not hold either: the flankers could not have yielded an effect of orthographic overlap without concurrent processing of the target.
